# Supplementary material for: Leaf-level coordination principles propagate to the ecosystem scale
Source: Nat Commun. 2023 Jul 4;14:3948. doi: 10.1038/s41467-023-39572-5 (PMC10319885; doi:10.1038/s41467-023-39572-5)
Supplement: Supplementary file 1 — Supplementary Information [file 41467_2023_39572_MOESM1_ESM.pdf]

Leaf-level coordination principles propagate to the ecosystem scale

Supplementary Tables

Supplementary Table 1. Leaf- and plant-level trait and other variables (left) and corresponding ecosystem analogues in our study (centre), with abbreviations (right).

Plant trait	Analogous Ecosystem Property	Abbreviation
Photosynthetic capacity (Amass)	Photosynthetic capacity at light saturation	GPPsat
Dark respiration rate (Rmass)	Maximum ecosystem respiration	RECOmax
Leaf longevity (LL)	Community-weighted mean leaf longevity	wLL
Nitrogen content per unit leaf mass (Nmass) (or N concentration)	Community-weighted mean nitrogen per unit leaf mass (or N concentration)	wNmass
Leaf area (LA)	Maximum leaf area index	LAImax
Plant height (H)	Maximum canopy height	Hc
Stem specific density (SSD)	Community-weighted mean stem specific density	wSSD
Stomatal conductance (gs)	Maximum surface conductance	Gsmax
Photosynthetic nitrogen use-efficiency (PNUE)	Photosynthetic nitrogen use-efficiency	PNUE
Water use-efficiency (WUE)	Water use-efficiency based on transpiration	WUEt
Air temperature (Ta)	Air temperature	Ta

Supplementary Table 2. Summary of linear mixed model calculated with the *lmer* function in the lme4[1] R Package, using the formula: *y ~ wNmass + wLMA + wLL + (LAImax | IGBP)*, where *y* stands for the predicted variable (GPPsat or RECOmax), on 87 observations (sites).

GPPsat prediction						
Random effects						
Groups	Name	Variance	Std.Dev.	Corr		
IGBP (n = 10)	(Intercept)	0.53	0.72			
	LAImax	1.90	1.38	1.00		
	Residual	25.30	5.03			
Fixed effects						
				Anova (Type II Wald chisquare tests)		
	Estimate	Std. Error	t value	Chisq	Df	Pr(>Chisq)
(Intercept)	10.63	3.60	2.95			
wNmass	6.07	1.72	3.52	12.38	1	0.000
wLMA	-13.44	11.30	-1.19	1.42	1	0.234
wLL	-0.06	0.03	-1.65	2.72	1	0.099
Correlation of Fixed Effects						
	(Intr)	wNmass	wLMA			
wNmass	-0.80					
wLMA	-0.58	0.17				
wLL	-0.27	0.26	-0.31			
RECOmax prediction						
Random effects						
Groups	Name	Variance	Std.Dev.	Corr		
IGBP (n = 10)	(Intercept)	0.42	0.65			
	LAImax	0.02	0.14	1.00		
	Residual	2.78	1.67			
Fixed effects						
				Anova (Type II Wald chisquare tests)		
	Estimate	Std. Error	t value	Chisq	Df	Pr(>Chisq)
(Intercept)	4.71	1.19	3.96			
wNmass	1.34	0.54	2.47	6.09	1	0.014
wLMA	-11.51	3.64	-3.16	9.99	1	0.002
wLL	0.00	0.01	-0.11	0.01	1	0.915
Correlation of Fixed Effects						
	(Intr)	wNmass	wLMA			
wNmass	-0.83					
wLMA	-0.58	0.23				
wLL	-0.27	0.26	-0.26			

Supplementary Table 3. Percentage of sites for each vegetation type for each section of the analysis. Abbreviations: IGBP: International Geosphere-Biosphere Programme vegetation type; CSH: closed shrubland; DBF: deciduous broadleaf forest; EBF: evergreen broadleaf forest; ENF: evergreen needleleaf forest; GRA: grassland; MF: mixed forest; OSH: open shrubland; SAV: savannah; WET: wetland; WSA: woody savannah.

IGBP	Leaf economics spectrum at the ecosystem scale	Ecosystem global spectrum of plant form and function	Least-cost hypothesis at the ecosystem scale
CSH	3.3	3.4	4.9
DBF	14.4	14.6	13.4
EBF	6.7	7.9	4.9
ENF	50.0	51.7	50.0
GRA	8.9	5.6	7.3
MF	5.6	5.6	4.9
OSH	2.2	2.2	3.7
SAV	2.2	3.4	3.7
WET	4.4	3.4	4.9
WSA	2.2	2.2	2.4

Supplementary Table 4. List of original FLUXNET variables used in the analysis.

Variable	Original variable name in FLUXNET 2015 dataset	Units	Variable name
GPP	GPP_NT_VUT_USTAR50	µmol CO_2 m^{-2} s^{-1}	Gross primary productivity from night-time partitioning
H	H_F_MDS	W m^{-2}	Sensible heat flux
LE	LE_F_MDS	W m^{-2}	Latent heat flux
NEE	NEE_NT_VUT_USTAR50	µmol CO_2 m^{-2} s^{-1}	Net ecosystem exchange
P	P	mm	Precipitation
RECO	RECO_NT_VUT_USTAR50	µmol CO_2 m^{-2} s^{-1}	Ecosystem respiration from night-time partitioning
SW_IN	SW_IN_F_MDS	W m^{-2}	Shortwave radiation, incoming, gap-filled,
TA	TA_F_MDS	° C	Air temperature
VPD	VPD_F_MDS	hPa	Vapour pressure deficit
USTAR	USTAR	m s^{-1}	Friction velocity
WS	WS	m s^{-1}	Wind speed

Supplementary Figures

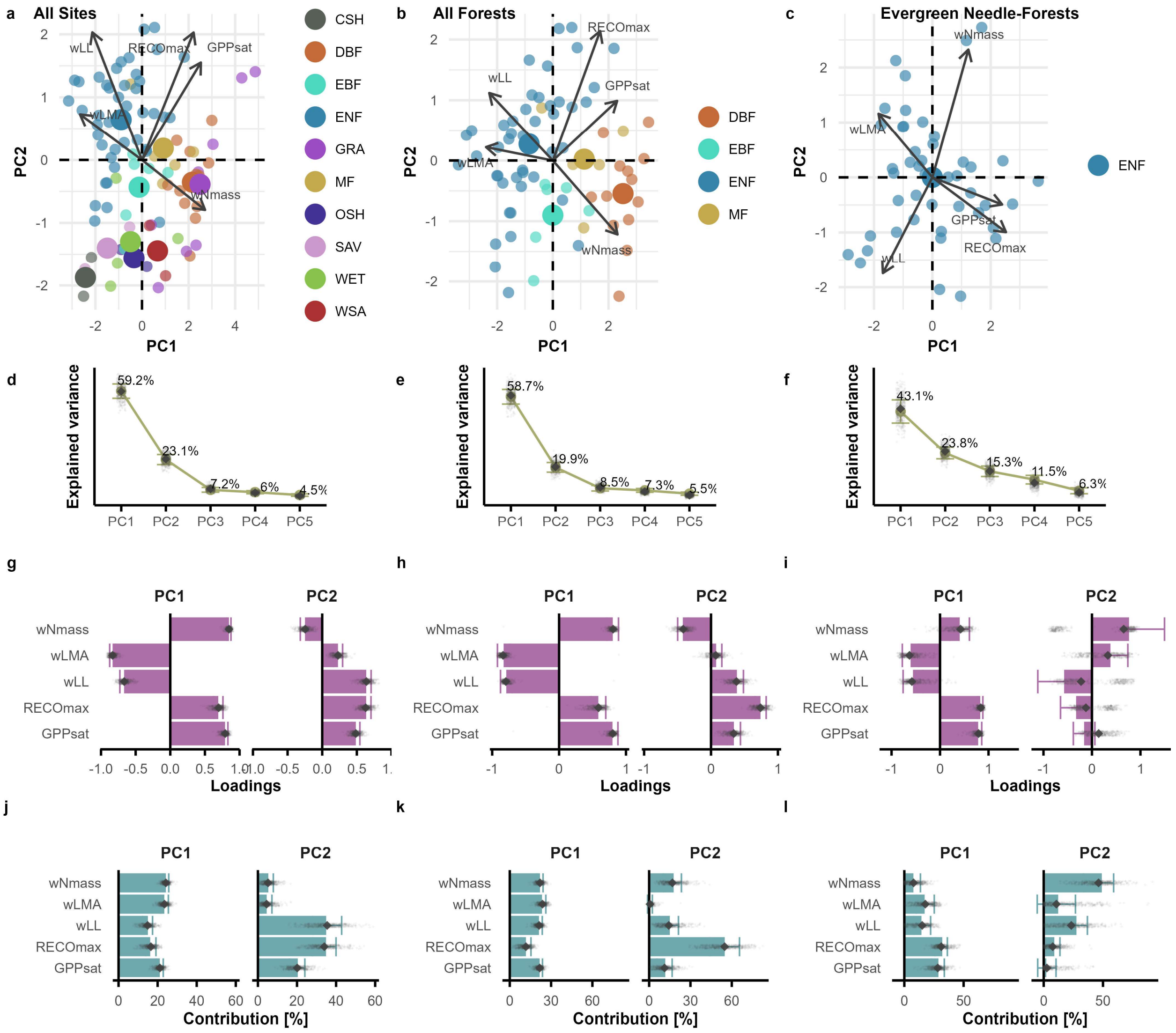

Supplementary Fig. 1. Principal Component Analysis (PCA) on variables representing the Leaf Economics Spectrum at the ecosystem scale on all sites (a, d, g, j, 90 sites), forest sites (b, e, h, k, 69 sites), and only evergreen needleleaf forests (c, f, i, l, 45 sites). For better comparison, we kept the same number of principal components as in the main analysis with all sites. **a, b, c** Biplot resulting from PCA; point colours represent plant functional types following the IGBP classification: CSH (Closed Shrubland), DBF (Deciduous Broadleaf Forest), EBF (Evergreen Broadleaf Forest), ENF (Evergreen Needleleaf Forest), GRA (Grassland), MF (Mixed Forest), OSH (Open Shrubland), SAV (Savannah), WET (Wetland), WSA (Woody Savannah).

Bigger points represent the centroid of the distribution for each habitat type. **d, e, f** Explained variance for the retained principal components (PCs). **g, h, i** Barplot for the loadings, and **j, k, l** contributions for each variable on the retained PCs. The full circles in d-f and the bars in g-l show the pertinent estimate based on the full dataset. In d-l the error bars are centred on the estimates and represent the standard error estimated with bootstrap procedure (n = 499 bootstrap iterations); the small grey diamonds show the estimates of each bootstrap iteration, and the big grey diamonds represent the median of all bootstrap iteration. Variable acronyms: photosynthetic capacity at light saturation (GPPsat), maximum ecosystem respiration (RECOmax), community-weighted mean leaf longevity (wLL), community-weighted mean leaf mass per area (wLMA), community-weighted mean nitrogen per leaf mass (wNmass).

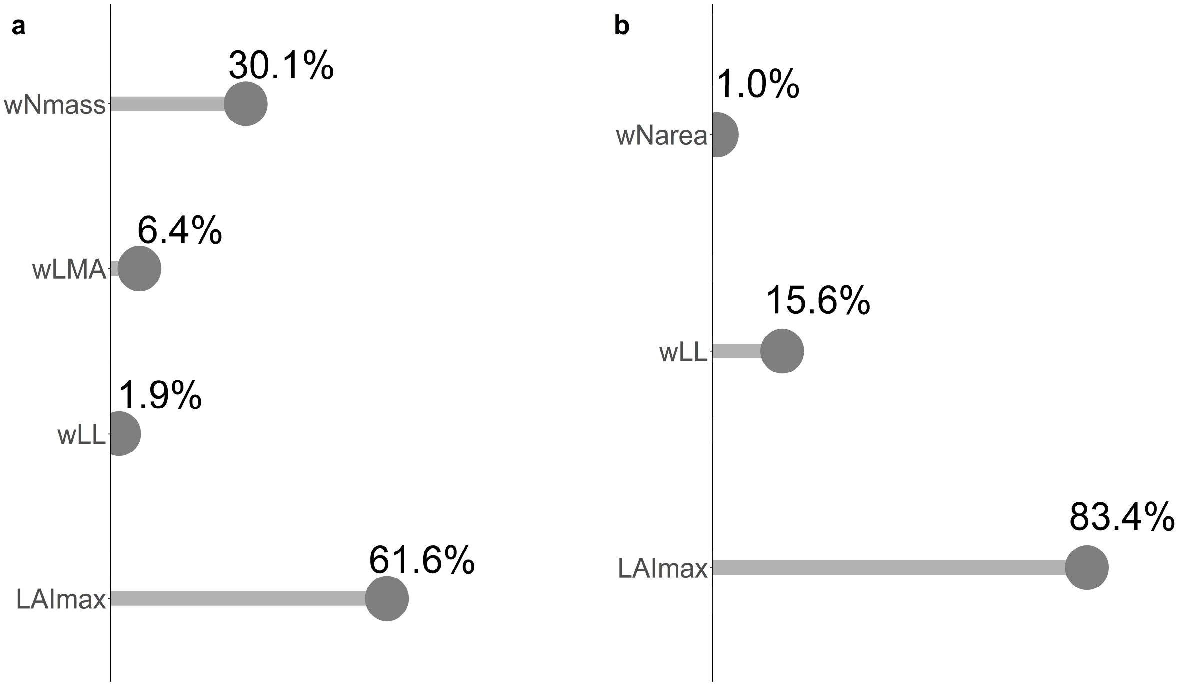

Supplementary Fig. 2. Relative importance[2] of the predictors of maximum Gross Primary Productivity at light saturation (GPPsat) - the ecosystem scale photosynthetic capacity, based on multimodel inference analysis[3] (n = 87). In panel **a** the analysis includes community-weighted mean N concentration (wNmass), community-weighted mean leaf mass per area (wLMA), community-weighted mean leaf longevity (wLL), and maximum leaf area index (LAImax) (62 % explained variance, mean Akaike's Information Criterion - AICc = 551.5). **b** Same as in panel a) but with area-based nitrogen estimates, which is wNmass divided by wLMA. Results show that LAImax followed by N content and average leaf longevity are the three most important predictors of the GPPsat (53 % explained variance, mean AICc = 568.7). Variable acronyms: community-weighted mean leaf longevity (wLL), community-weighted mean leaf mass per area (wLMA), community-weighted mean nitrogen per leaf area (wNarea), community-weighted mean nitrogen per leaf mass (wNmass), maximum leaf area index (LAImax).

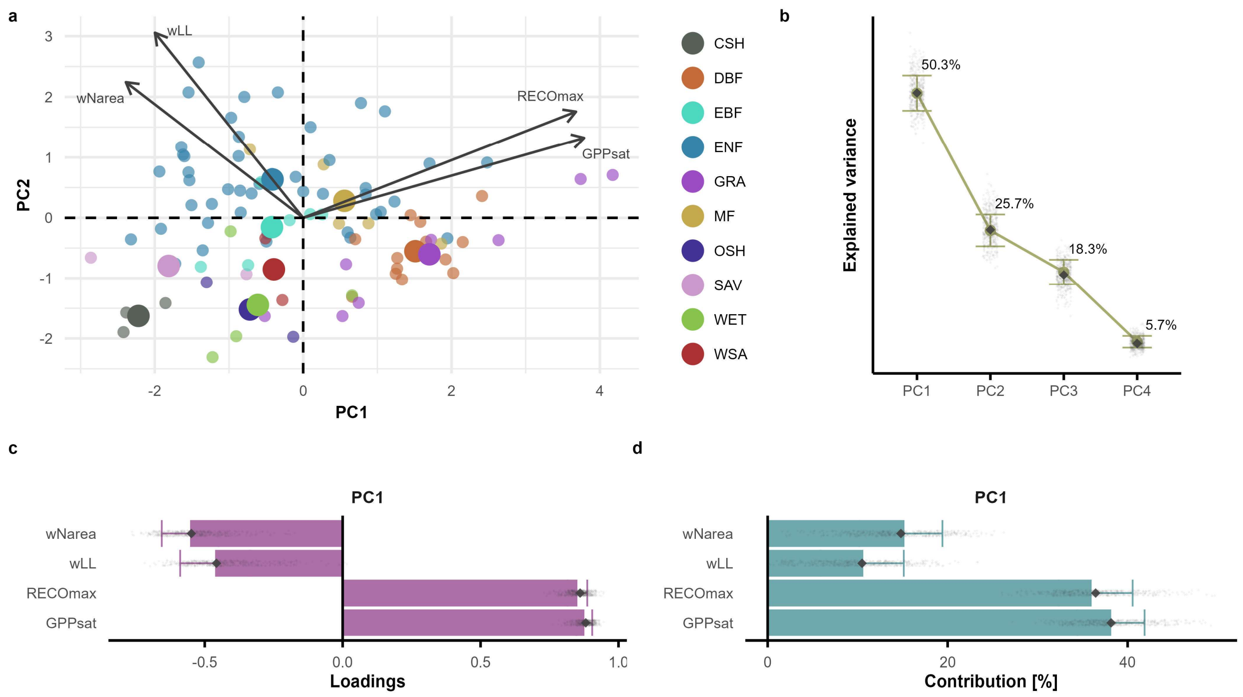

Supplementary Fig. 3. Principal Component Analysis (PCA) on leaf economics spectrum analogue with wNarea at the ecosystem scale (90 sites). a Biplot resulting from PCA; point colours represent plant functional types following the IGBP classification: CSH (Closed Shrubland), DBF (Deciduous Broadleaf Forest), EBF (Evergreen Broadleaf Forest), ENF (Evergreen Needleleaf Forest), GRA (Grassland), MF (Mixed Forest), OSH (Open Shrubland), SAV (Savannah), WET (Wetland), WSA (Woody Savannah). Bigger points represent the centroid of the distribution for each vegetation type. **b** Explained variance for the retained principal components (PCs). **c** Barplot for the loadings, and **d** contributions for each variable on the retained PCs. The full circles in b and the bars in c and d show the pertinent estimate based on the full dataset. In b, c, and d, the error bars are centred on the estimates and represent the standard error estimated with bootstrap procedure (n = 499 bootstrap iterations); the small grey diamonds show the estimates of each bootstrap iteration, and the big grey diamonds represent the median of all bootstrap iteration. Variable acronyms: gross primary productivity at light saturation (GPPsat), maximum ecosystem respiration (RECOmax), community-weighted mean leaf longevity (wLL), community-weighted mean nitrogen per leaf area (wNarea).

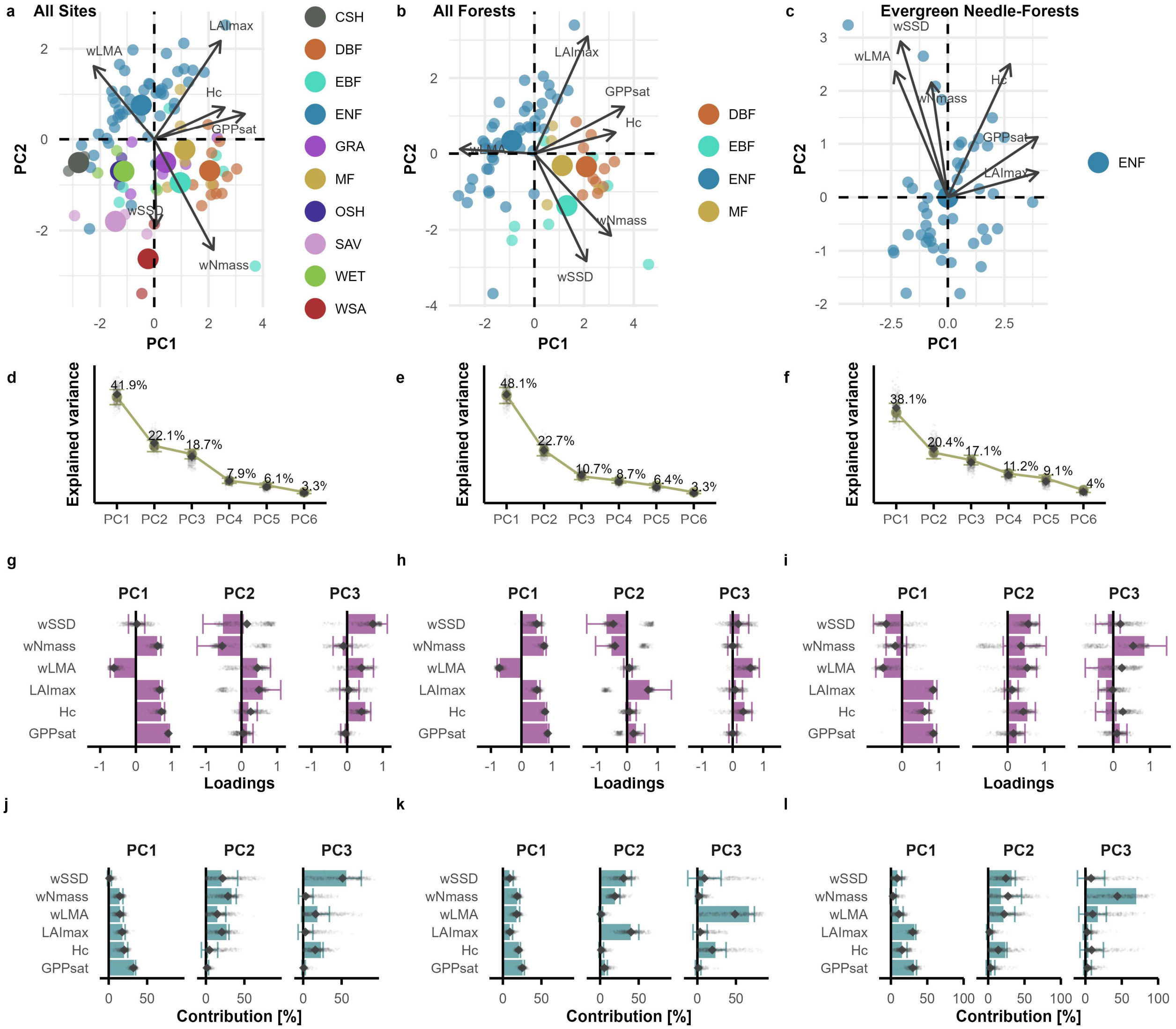

Supplementary Fig. 4. Principal Component Analysis on the Global Spectrum of Plant Form and Function at the ecosystem scale on all sites (a, d, g, j, 89 sites), forest sites (b, e, h, k, 71 sites), and only evergreen needleleaf forests (c, f, i, l, 46 sites). For better comparison, we kept the same number of principal components as in the main analysis with all sites. **a, b, c** Biplot resulting from PCA; point colours represent plant functional types following the IGBP classification: CSH (Closed Shrubland), DBF (Deciduous Broadleaf Forest), EBF (Evergreen Broadleaf Forest), ENF (Evergreen Needleleaf Forest), GRA (Grassland), MF (Mixed Forest), OSH (Open Shrubland), SAV (Savannah), WET (Wetland), WSA (Woody Savannah). Bigger points represent the centroid of the distribution for each habitat type. **d, e, f** Explained variance for the retained

principal components (PCs). **g, h, i** Barplot for the loadings, and **j, k, l** contributions for each variable on the retained PCs. The full circles in d-f and the bars in g-l show the pertinent estimate based on the full dataset. In d-l the error bars are centred on the estimates and represent the standard error estimated with bootstrap procedure (n = 499 bootstrap iterations); the small grey diamonds show the estimates of each bootstrap iteration, and the big grey diamonds represent the median of all bootstrap iteration. Variable acronyms: gross primary productivity at light saturation (GPPsat), canopy height (Hc), maximum leaf area index (LAImax), community-weighted mean leaf mass per area (wLMA), community-weighted mean nitrogen per leaf mass (wNmass), community-weighted mean stem specific density (wSSD).

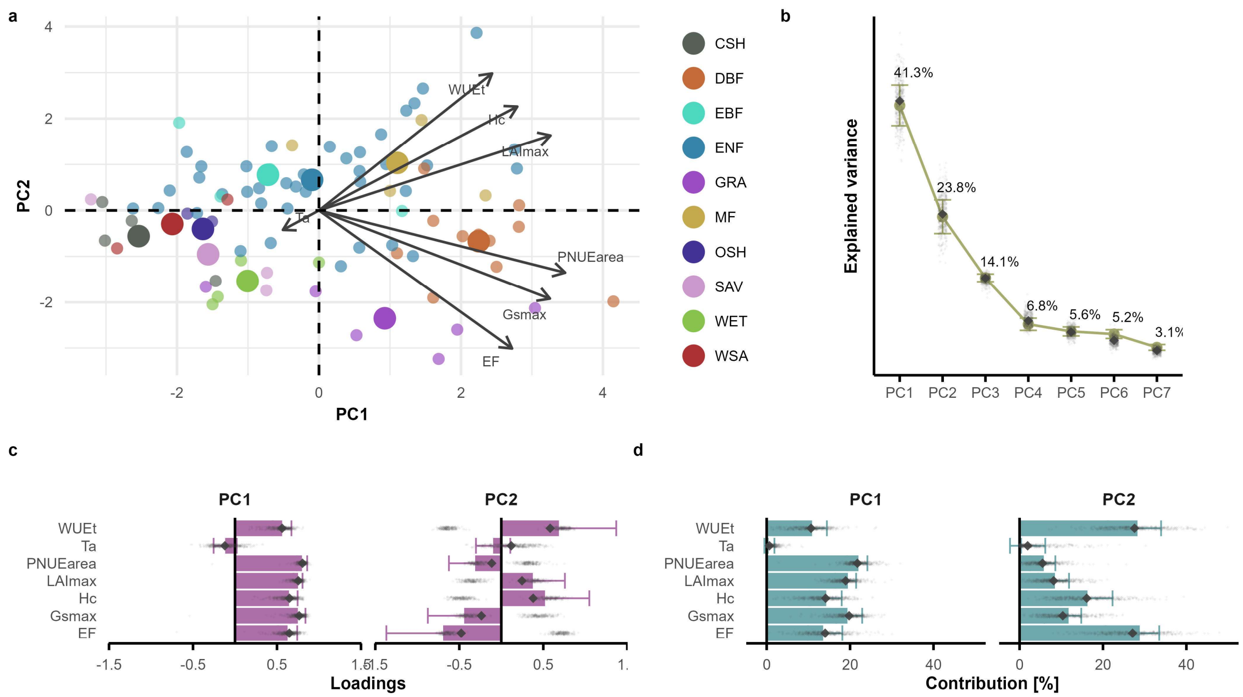

Supplementary Fig. 5. Principal Component Analysis (PCA) on least-cost hypothesis analogue with PNUEarea and LAImax at the ecosystem scale (82 sites). a Biplot resulting from PCA; point colours represent plant functional types following the IGBP classification: CSH (Closed Shrubland), DBF (Deciduous Broadleaf Forest), EBF (Evergreen Broadleaf Forest), ENF (Evergreen Needleleaf Forest), GRA (Grassland), MF (Mixed Forest), OSH (Open Shrubland), SAV (Savannah), WET (Wetland), WSA (Woody Savannah). Bigger points represent the centroid of the distribution for each vegetation type. **b** Explained variance for the retained principal components (PCs). **c** Barplot for the loadings, and **d** contributions for each variable on the retained PCs. The full circles in b and the bars in c and d show the pertinent estimate based on the full dataset. In b, c, and d, the error bars are centred on the estimates and represent the standard error estimated with bootstrap procedure (n = 499 bootstrap iterations); the small grey diamonds show the estimates of each bootstrap iteration, and the big grey diamonds represent the median of all bootstrap iteration. Variable acronyms: evaporative fraction (EF), maximum surface conductance (Gsmax), canopy height (Hc), maximum leaf area index (LAImax), photosynthetic nitrogen use efficiency without LAImax

normalization (PNUEarea), air temperature (Ta), water use efficiency based on transpiration (WUEt). We calculated PNUEarea based on Narea, without normalizing by LAImax (PNUEarea = GPPsat / wNarea).

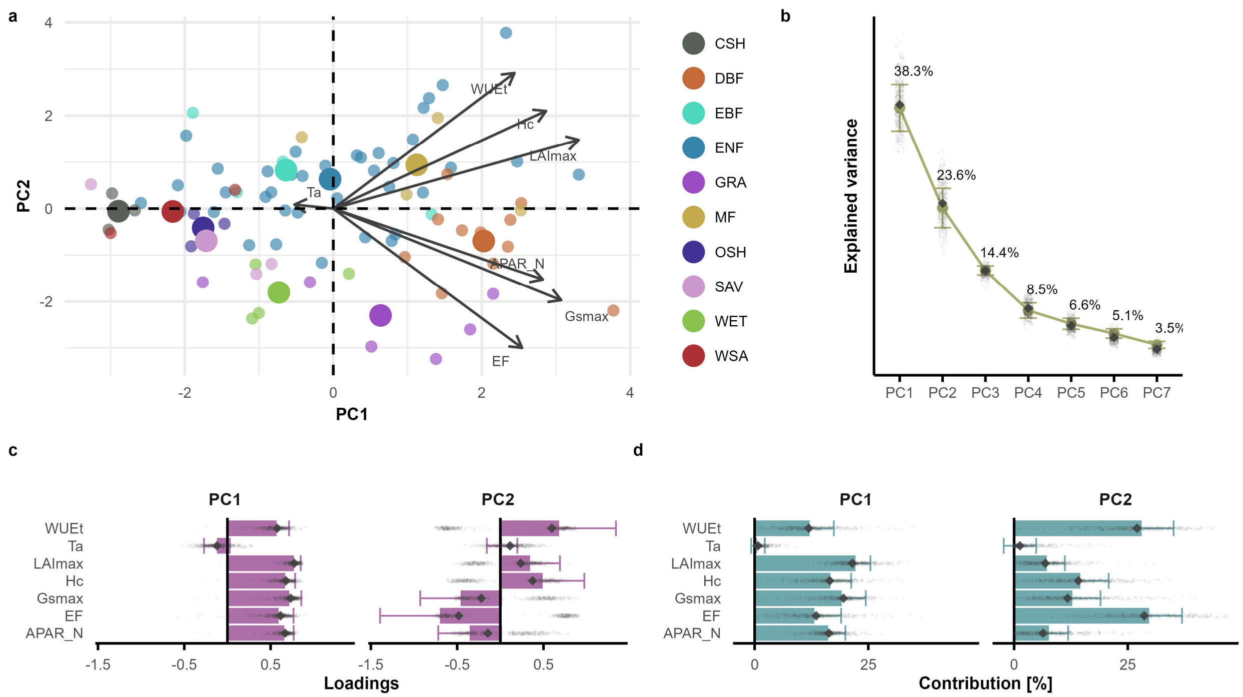

Supplementary Fig. 6. Principal Component Analysis (PCA) on least-cost hypothesis analogue with APAR_N and LAImax at the ecosystem scale (80 sites). a Biplot resulting from PCA; point colours represent plant functional types following the IGBP classification: CSH (Closed Shrubland), DBF (Deciduous Broadleaf Forest), EBF (Evergreen Broadleaf Forest), ENF (Evergreen Needleleaf Forest), GRA (Grassland), MF (Mixed Forest), OSH (Open Shrubland), SAV (Savannah), WET (Wetland), WSA (Woody Savannah). Bigger points represent the centroid of the distribution for each vegetation type. **b** Explained variance for the retained principal components (PCs). **c** Barplot for the loadings, and **d** contributions for each variable on the retained PCs. The full circles in b and the bars in c and d show the pertinent estimate based on the full dataset. In b, c, and d, the error bars are centred on the estimates and represent the standard error estimated with bootstrap procedure (n = 499 bootstrap iterations); the small grey diamonds show the estimates of each bootstrap iteration, and the big grey diamonds represent the median of all bootstrap iteration. Variable acronyms: ratio between maximum absorbed photosynthetically active radiation and community-weighted mean of nitrogen per leaf mass (APAR_N), evaporative fraction (EF), maximum surface conductance (Gsmax), canopy height (Hc), maximum leaf area index (LAImax), air temperature

(Ta), water use efficiency based on transpiration (WUEt). To avoid a spurious relationship between PNUE and WUE, we developed the alternative metric of APAR_N, related to PNUE, and based on absorbed photosynthetically active radiation (APAR), since APAR correlates with GPPsat. We computed APAR as the product between fPAR, and the photosynthetic photon flux density (PPFD, µmol m-2 s-1) of photosynthetically active radiation. We first extracted data for the fraction of photosynthetically active radiation (fPAR, -) from the MCD15A3H MODIS product. For each flux tower location, we averaged the fPAR information from an area of 3 x 3 500 m^2 pixels. We used the fPAR-LAI quality information provided in the MCD15A3H product flags to focus on data points retrieved from the main algorithm under the following criteria: good retrieval quality, with and without saturation, limited dead detector problems, and clear sky conditions. For a more detailed filtering, we removed ocean or shore pixels, snow or ice, as well as clouds, cloud shadows and cirrus. After aggregating SW_IN to 4 days to match the temporal aggregation of the extracted fPAR, we multiplied SW_IN by 2.11 for the conversion to PPFD. We calculated the yearly APAR as the 90th percentile of 4-days aggregates of the product between fPAR and PPFD, and averaged yearly APAR values to site means. APAR was then computed as the mean yearly maximum at each site. We computed APAR_N = APARmax / wNarea as an independent proxy of “potential” PNUE.

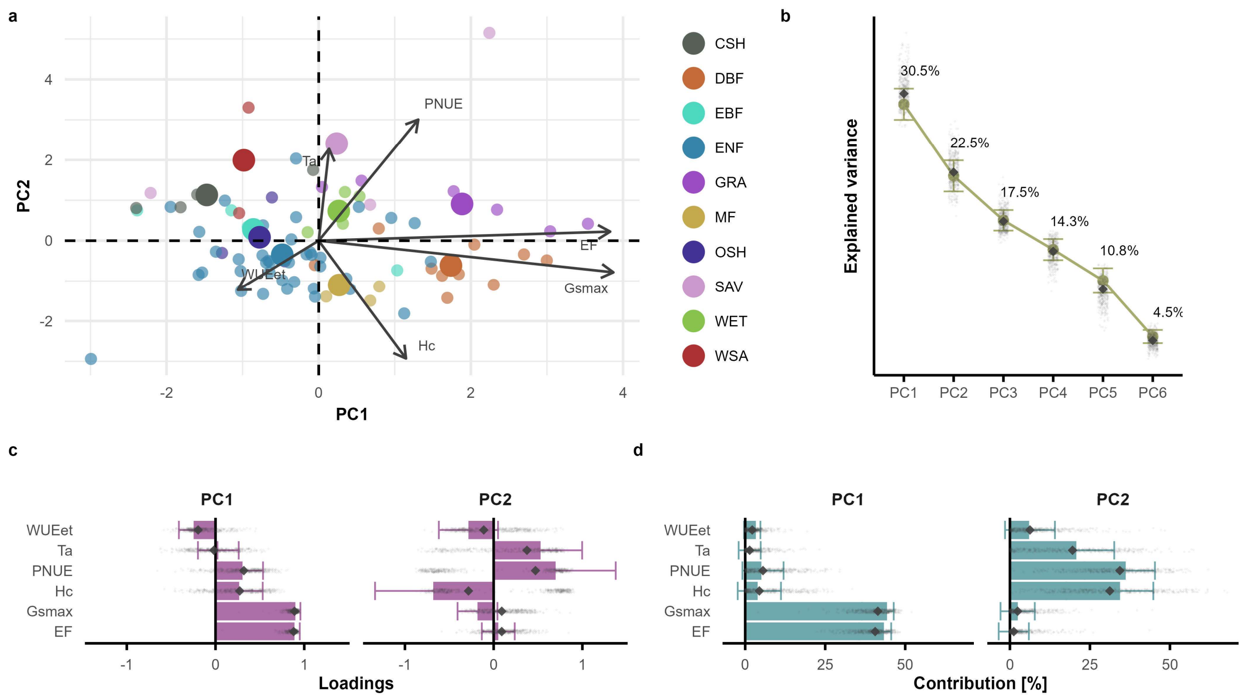

Supplementary Fig. 7. Principal Component Analysis (PCA) on least-cost hypothesis analogue with WUE based on evapotranspiration (WUEet) at the ecosystem scale (82 sites). **a** Biplot resulting from PCA; point colours represent plant functional types following the IGBP classification: CSH (Closed Shrubland), DBF (Deciduous Broadleaf Forest), EBF (Evergreen Broadleaf Forest), ENF (Evergreen Needleleaf Forest), GRA (Grassland), MF (Mixed Forest), OSH (Open Shrubland), SAV (Savannah), WET (Wetland), WSA (Woody Savannah). Bigger points represent the centroid of the distribution for each vegetation type. **b** Explained variance for the retained principal components (PCs). **c** Barplot for the loadings, and **d** contributions for each variable on the retained PCs. The full circles in b and the bars in c and d show the pertinent estimate based on the full dataset. In b, c, and d, the error bars are centred on the estimates and represent the standard error estimated with bootstrap procedure (n = 499 bootstrap iterations); the small grey diamonds show the estimates of each bootstrap iteration, and the big grey diamonds represent the median of all bootstrap iteration. Variable acronyms: evaporative fraction (EF), maximum surface conductance (Gsmax), canopy height (Hc), photosynthetic nitrogen use efficiency (PNUE), air temperature (Ta), water use efficiency based on evapotranspiration (WUEet).

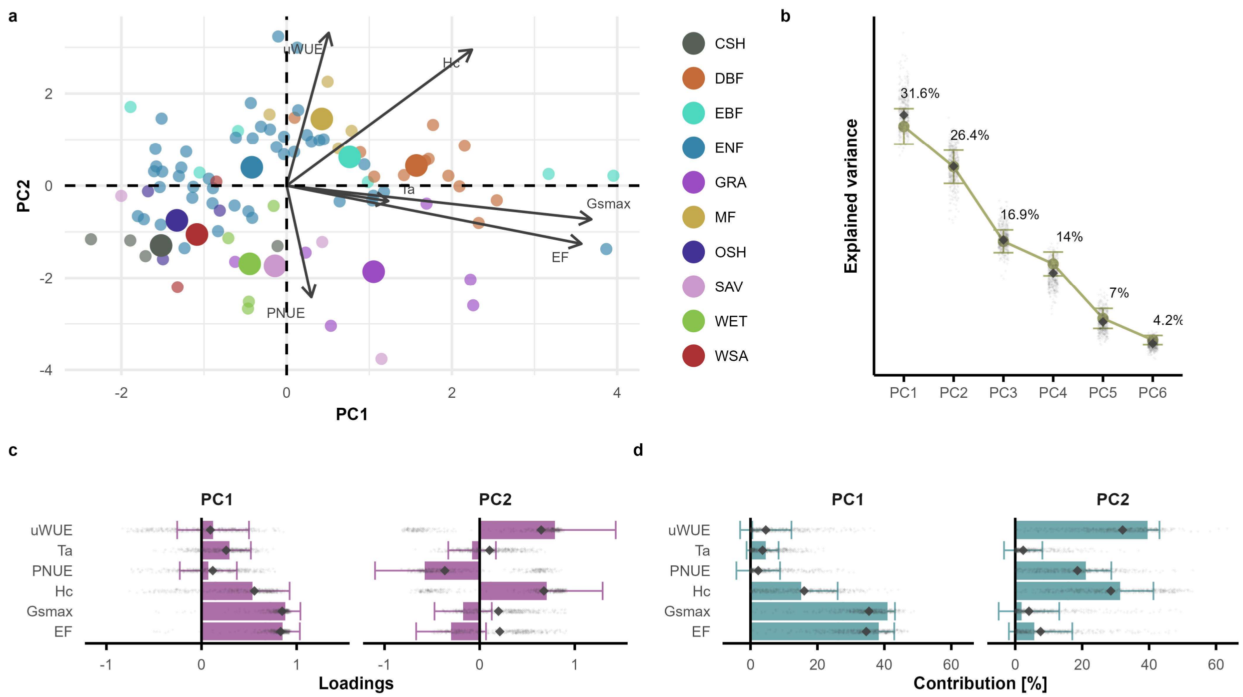

Supplementary Fig. 8. Principal Component Analysis (PCA) on least-cost hypothesis analogue with the underlying water use-efficiency (uWUE)[4] at the ecosystem scale (89 sites). a Biplot resulting from PCA; point colours represent plant functional types following the IGBP classification: CSH (Closed Shrubland), DBF (Deciduous Broadleaf Forest), EBF (Evergreen Broadleaf Forest), ENF (Evergreen Needleleaf Forest), GRA (Grassland), MF (Mixed Forest), OSH (Open Shrubland), SAV (Savannah), WET (Wetland), WSA (Woody Savannah). Bigger points represent the centroid of the distribution for each vegetation type. **b** Explained variance for the retained principal components (PCs). **c** Barplot for the loadings, and **d** contributions for each variable on the retained PCs. The full circles in b and the bars in c and d show the pertinent estimate based on the full dataset. In b, c, and d, the error bars are centred on the estimates and represent the standard error estimated with bootstrap procedure (n = 499 bootstrap iterations); the small grey diamonds show the estimates of each bootstrap iteration, and the big grey diamonds represent the median of all bootstrap iteration. Variable acronyms: evaporative fraction (EF), maximum surface conductance (Gsmax), canopy height (Hc), photosynthetic nitrogen use efficiency (PNUE), air temperature (Ta), underlying water use efficiency (uWUE).

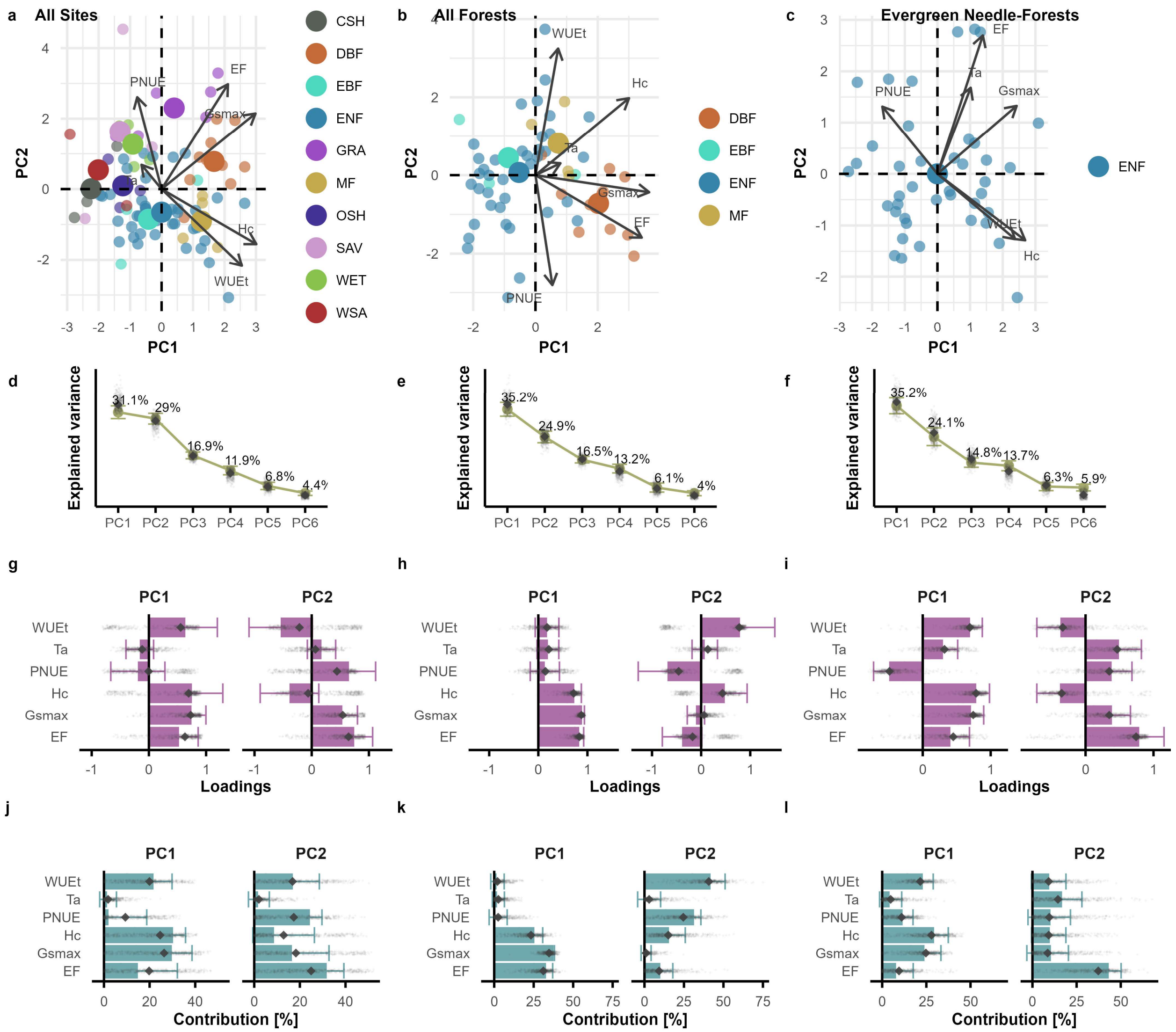

Supplementary Fig. 9. Principal Component Analysis (PCA) on the Least-Cost Hypothesis at the ecosystem scale on all sites (a, d, g, j, 82 sites), forest sites (b, e, h, k, 60 sites), and only evergreen needleleaf forests (c, f, i, l, 41 sites). For better comparison, we kept the same number of principal components as in the main analysis with all sites. **a, b, c** Biplot resulting from PCA; point colours represent plant functional types following the IGBP classification: CSH (Closed Shrubland), DBF (Deciduous Broadleaf Forest), EBF (Evergreen Broadleaf Forest), ENF (Evergreen Needleleaf Forest), GRA (Grassland), MF (Mixed Forest), OSH (Open Shrubland), SAV (Savannah), WET (Wetland), WSA (Woody Savannah). Bigger points represent the centroid of the distribution for each habitat type. **d, e, f** Explained variance for the retained

principal components (PCs). **g, h, i** Barplot for the loadings, and **j, k, l** contributions for each variable on the retained PCs. The full circles in d-f and the bars in g-l show the pertinent estimate based on the full dataset. In d-l the error bars are centred on the estimates and represent the standard error estimated with bootstrap procedure (n = 499 bootstrap iterations); the small grey diamonds show the estimates of each bootstrap iteration, and the big grey diamonds represent the median of all bootstrap iteration. Variable acronyms: evaporative fraction (EF), maximum surface conductance (Gsmax), canopy height (Hc), photosynthetic nitrogen use efficiency (PNUE), air temperature (Ta), water use efficiency based on transpiration (WUEt).

References

1. Bates, D., Mächler, M., Bolker, B. & Walker, S. Fitting Linear Mixed-Effects Models Using lme4. *Journal of Statistical Software* **67**, 1–48 (2015).
2. Grömping, U. Relative Importance for Linear Regression in R: The Package relaimpo. *Journal of Statistical Software* **17**, 1–27 (2006).
3. Bartoń, K. MuMIn: Multi-modal inference. Model selection and model averaging based on information criteria (AICc and alike). *http://cran.r-project.org/web/packages/MuMIn/index.html* (2013).
4. Zhou, S., Yu, B., Huang, Y. & Wang, G. The effect of vapor pressure deficit on water use efficiency at the subdaily time scale. *Geophysical Research Letters* **41**, 5005–5013 (2014).
