# Supplementary material for: Leaf-level coordination principles propagate to the ecosystem scale
Source: Nat Commun. 2023 Jul 4;14:3948. doi: 10.1038/s41467-023-39572-5 (PMC10319885; doi:10.1038/s41467-023-39572-5)
Supplement: Supplementary file 8 — Reporting Summary [file 41467_2023_39572_MOESM8_ESM.pdf]

nature portfolio

Corresponding author(s): Ulisse Gomarasca

Last updated by author(s): May 30, 2023

Reporting Summary

Nature Portfolio wishes to improve the reproducibility of the work that we publish. This form provides structure for consistency and transparency in reporting. For further information on Nature Portfolio policies, see our Editorial Policies and the Editorial Policy Checklist.

Statistics

For all statistical analyses, confirm that the following items are present in the figure legend, table legend, main text, or Methods section.

n/a	Confirmed	
☐	☒	The exact sample size (*n*) for each experimental group/condition, given as a discrete number and unit of measurement
☐	☒	A statement on whether measurements were taken from distinct samples or whether the same sample was measured repeatedly
☐	☒	The statistical test(s) used AND whether they are one- or two-sided<br>*Only common tests should be described solely by name; describe more complex techniques in the Methods section.*
☐	☒	A description of all covariates tested
☐	☒	A description of any assumptions or corrections, such as tests of normality and adjustment for multiple comparisons
☐	☒	A full description of the statistical parameters including central tendency (e.g. means) or other basic estimates (e.g. regression coefficient) AND variation (e.g. standard deviation) or associated estimates of uncertainty (e.g. confidence intervals)
☐	☒	For null hypothesis testing, the test statistic (e.g. *F*, *t*, *r*) with confidence intervals, effect sizes, degrees of freedom and *P* value noted<br>*Give P values as exact values whenever suitable.*
☒	☐	For Bayesian analysis, information on the choice of priors and Markov chain Monte Carlo settings
☒	☐	For hierarchical and complex designs, identification of the appropriate level for tests and full reporting of outcomes
☒	☐	Estimates of effect sizes (e.g. Cohen's *d*, Pearson's *r*), indicating how they were calculated

Our web collection on statistics for biologists contains articles on many of the points above.

Software and code

Policy information about availability of computer code

Data collection	No software was used for data collection as the datasets were available in online repositories.
Data analysis	All the analyses were conducted with R 4.1.0 for Windows (64-bit). The R package used for the calculation of the ecosystem functional properties is already described in the literature and freely available on CRAN: bigleaf v0.8.2 (https://cran.r-project.org/web/packages/bigleaf/). The R code used for the statistical analyses uses packages available on CRAN: FactoMineR v2.6 (https://cran.r-project.org/web/packages/FactoMineR/), ade4 v1.7-20 (https://cran.r-project.org/web/packages/ade4/), modelr v0.1.9 (https://cran.r-project.org/web/packages/modelr/), MuMIn v1.43.17 (https://cran.r-project.org/web/packages/MuMIn/), relaimpo v2.2-6 (https://cran.r-project.org/web/packages/relaimpo/), and lme4 v1.1-31 (https://cran.r-project.org/web/packages/lme4/). The TEA algorithm v1.1 is available at https://doi.org/10.5281/zenodo.3921923. The R codes used for this analysis are available on zenodo at https://doi.org/10.5281/zenodo.7984734.

For manuscripts utilizing custom algorithms or software that are central to the research but not yet described in published literature, software must be made available to editors and reviewers. We strongly encourage code deposition in a community repository (e.g. GitHub). See the Nature Portfolio guidelines for submitting code & software for further information.

Data

Policy information about availability of data

All manuscripts must include a data availability statement. This statement should provide the following information, where applicable:

- Accession codes, unique identifiers, or web links for publicly available datasets
- A description of any restrictions on data availability
- For clinical datasets or third party data, please ensure that the statement adheres to our policy

The processed eddy-covariance data – the LaThuile dataset (https://fluxnet.fluxdata.org/data/la-thuile-dataset/) and the FLUXNET2015 dataset (https://fluxnet.fluxdata.org/data/fluxnet2015-dataset/) – are available on the FLUXNET website. Biological, Ancillary, Disturbance and Metadata for the sites are available in the respective databases (https://fluxnet.org/data/fluxnet2015-dataset/, https://fluxnet.org/data/la-thuile-dataset/, https://ameriflux.lbl.gov/data/badm/, https://ameriflux.lbl.gov/sites/site-search/, and https://www.ozflux.org.au/monitoringsites/calperum/calperum_dem.html) and in the cited literature. The plant traits measurements data are available on the TRY database (https://www.try-db.org/TryWeb/Home.php) either publicly or under restricted access due to embargo, and can be obtained via request on the TRY platform. The data necessary to interpret, verify, and extend the research in this article are available in the zenodo database under accession code https://doi.org/10.5281/zenodo.7984734.

Human research participants

Policy information about studies involving human research participants and Sex and Gender in Research.

Reporting on sex and gender	n.a.
Population characteristics	n.a.
Recruitment	n.a.
Ethics oversight	n.a.

Note that full information on the approval of the study protocol must also be provided in the manuscript.

Field-specific reporting

Please select the one below that is the best fit for your research. If you are not sure, read the appropriate sections before making your selection.

☐ Life sciences ☐ Behavioural & social sciences ☒ Ecological, evolutionary & environmental sciences

For a reference copy of the document with all sections, see nature.com/documents/nr-reporting-summary-flat.pdf

Ecological, evolutionary & environmental sciences study design

All studies must disclose on these points even when the disclosure is negative.

Study description

We tested three well-known leaf- and plant-level coordination theories at the ecosystem scale. We derive ecosystem functional properties from a global dataset (98 sites) of surface gas exchange measurements, and vegetation properties from site measurements or plant trait databases.

Research sample

The data used for the calculation of ecosystem functional properties belongs to the FLUXNET La Thuile and FLUXNET2015 Tier 1 and Tier 2 dataset, the global network of CO2, water and energy flux measurements. The sites used cover a wide variety of climate zones (from tropical to arctic) and vegetation types (wetlands, shrublands, savannahs, grasslands, evergreen and deciduous forests boreal, temperate and tropical forests). The FLUXNET LaThuile is available at: https://fluxnet.fluxdata.org/data/la-thuile-dataset/. FLUXNET2015 is available at https:// fluxnet.fluxdata.org/data/fluxnet2015-dataset/. Information on the sites used and related sources are included in the supplementary materials (Supplementary Table 6).
Species information, and Biological, Ancillary, Disturbance and Metadata for the sites were collected from databases and literature, or from communication with site PIs.
Plant traits measurements were collected from the literature, from communication with site PIs, or from the TRY database at https://www.try-db.org/TryWeb/Home.php. Information on the trait datasets used and related sources are included in the supplementary materials (Supplementary Table 8).

Sampling strategy

We used data from the global network of eddy covariance flux tower stations (FLUXNET), integrating the LaThuile dataset (cf. ref. 46) with the FLUXNET2015 dataset (cf. ref. 47). In case of overlap of sites in the two datasets, the FLUXNET2015 dataset was used. We excluded cropland sites in order to avoid the influence of intense management practices (irrigation, ploughing, fertilization, etc.). The dataset used for the analysis included sites with more than 3 years of data and availability of ancillary data described below. The selected 98 sites cover different biomes and climate zones: from tropical, Mediterranean, temperate, and boreal to arctic sites, including major forest types, grasslands, savannas, shrublands, and wetlands (Supplementary Data 4, Supplementary Table 3). We retained data with good quality (quality check 0 – measured data, and 1 – good quality gap-filled data), and, additionally, we retained the data measured during the active growing season, determined as the period when daily GPP is above the 30% of the difference

between maximum and minimum daily GPP.
For each FLUXNET site we collected a set of plant traits for constituent species or site means (leaf longevity, leaf mass per area, nitrogen per leaf area, nitrogen per leaf mass, and stem specific density), and site-level vegetation characteristics (canopy height, maximum leaf area index) from the FLUXNET or Ameriflux ancillary data, or, if not reported, directly from site principal investigators. Where site measurements were unavailable, we included information from the TRY database (a full list of plant traits data sources can be found in Supplementary Data 5, cf. ref. 48), or data from the literature for the specific sites (refs. 30,49,50). We obtained site constituent species and species abundances at the sites (percentage of area covered by each species) from the literature (refs. 49–53), and by consulting site principal investigators. We assumed homogeneous distribution for species with missing abundance information, following the approach described in previous studies (refs. 51,52). We excluded sites where the total sum of known species abundances was below 50% of the total site area.

Data collection

Data for the calculation of ecosystem functional properties were recorded using the eddy covariance technique, which is based on a combination of a gas analyzer and ultrasonic anemometer associated with a meteorological station. FLUXNET is global network of site principle investigators and collaborators and processed with standardized procedures. Site PIs and collaborators are responsible for data collection for individual site eddy covariance measurements.
Plant traits data were collected from the TRY database, which is a network of vegetation scientists headed by Future Earth and the Max Planck Institute for Biogeochemistry. TRY integrates about 700 datasets and provides a global database of curated plant traits. PIs and collaborators are responsible for data collection of individual measurements or datasets.

Timing and spatial scale

The eddy-covariance data are half-hourly and we selected sites with at least 3 years of data. The start and end of measurements is different site by site and depends on the date of installation of the equipment. The sites used cover a wide variety of climate zones and vegetation types . The total number of sites is 98. Each site is representative of a spatial scale randing from ~200 m for grasslands to ~1 km for forests, depending on the measurements height.
Measurements of plant traits and vegetation properties are point measurements at specific locations, but were averaged to single estimates, where appropriate.

Data exclusions

From the original FLUXNET datasets we excluded croplands to avoid the inclusion of sites heavily managed in the analysis (e.g. fertilization, irrigation, etc). Sites were additionally excluded if they did not fullfil the filtering criteria described in the Method section, or if the measurement period was shorter than 3 years.
We excluded plant traits measurements conducted under experimental setups that would not represent natural ambient conditions (e.g. elevated CO2).

Reproducibility

We did not collect measurements directly but used widely documented global datasets and literature data. Data and codes the data necessary to reproduce the analysis are provided.

Randomization

Permutation and randomization were used to test the number of components to be retained with the Principal Component Analysis and the significance of the loadings to each component. Permutation and bootstrap was used to assess the statistical significance and the standard errors of the fittings presented in the analysis.

Blinding

Blinding was not needed in this study because we do not have treatments.

Did the study involve field work? ☐ Yes ☒ No

Reporting for specific materials, systems and methods

We require information from authors about some types of materials, experimental systems and methods used in many studies. Here, indicate whether each material, system or method listed is relevant to your study. If you are not sure if a list item applies to your research, read the appropriate section before selecting a response.

Materials & experimental systems

n/a	Involved in the study
☒	☐ Antibodies
☒	☐ Eukaryotic cell lines
☒	☐ Palaeontology and archaeology
☒	☐ Animals and other organisms
☒	☐ Clinical data
☒	☐ Dual use research of concern

Methods

n/a	Involved in the study
☒	☐ ChIP-seq
☒	☐ Flow cytometry
☒	☐ MRI-based neuroimaging
